# Functional activation of the periaqueductal gray matter during conditioned and unconditioned fear in guinea pigs confronted with the *Boa constrictor constrictor* snake

**DOI:** 10.1590/1414-431X2021e11542

**Published:** 2022-02-16

**Authors:** B.B. de Paula, E.B. Vieira-Rasteli, F. Calvo, N.C. Coimbra, C.R.A. Leite-Panissi

**Affiliations:** 1 Departamento de Psicologia, Faculdade de Filosofia, Ciências e Letras de Ribeirão Preto, Universidade de São Paulo, Ribeirão Preto, SP, Brasil; 2 Laboratório de Neuroanatomia e Neuropsicobiologia, Departamento de Farmacologia, Faculdade de Medicina de Ribeirão Preto, Universidade de São Paulo, Ribeirão Preto, SP, Brasil; 3 NAP-USP Centro de Pesquisas em Neurobiologia das Emoções, Faculdade de Medicina de Ribeirão Preto, Universidade de São Paulo, Ribeirão Preto, SP, Brasil; 4 Instituto de Neurociências e Comportamento, Ribeirão Preto, SP, Brasil; 5 Ophidiarium LNN-FMRP-USP/INeC, Departamento de Farmacologia, Faculdade de Medicina de Ribeirão Preto, Universidade de São Paulo, Ribeirão Preto, SP, Brasil

**Keywords:** Unconditioned fear, Conditioned fear, Periaqueductal gray matter, Prey *vs* snake paradigm, Boa constrictor constrictor

## Abstract

The periaqueductal gray matter (PAG) is an essential structure involved in the elaboration of defensive responses, such as when facing predators and conspecific aggressors. Using a prey *vs* predator paradigm, we aimed to evaluate the PAG activation pattern evoked by unconditioned and conditioned fear situations. Adult male guinea pigs were confronted either by a *Boa constrictor constrictor* wild snake or by the aversive experimental context. After the behavioral test, the rodents were euthanized and the brain prepared for immunohistochemistry for Fos protein identification in different PAG columns. Although Fos-protein-labeled neurons were found in different PAG columns after both unconditioned and conditioned fear situations at the caudal level of the PAG, we found greater activation of the lateral column compared to the ventrolateral and dorsomedial columns after predator exposure. Moreover, the lateral column of the PAG showed higher Fos-labeled cells at the caudal level compared to the same area at the rostral level. The present results suggested that there are different activation patterns of PAG columns during unconditioned and conditioned fear in guinea pigs. It is possible to hypothesize that the recruitment of specific PAG columns depended on the nature of the threatening stimulus.

## Introduction

The triggering of defensive behaviors involves the activation of a complex neural system responsible for the recognition and evaluation of the aversive stimuli and, ultimately, for appropriate motor activity to exert the most appropriate defensive response. The periaqueductal gray matter (PAG) has traditionally been considered the output station of the encephalic aversion system, stimulating the endogenous pain modulatory system (1) and spinal cord ventral horn motoneurons (2,3). Furthermore, it has been shown that aversive unconditioned stimuli produce a significant increase in the activity of limbic and paralimbic structures, such as the PAG, hypothalamus, amygdaloid complex, and corpora quadrigemina, which elicit defensive behavior that allow flight or attack (4-6). However, the organization of defensive responses seems to be hierarchically modulated, as responses induced by amygdaloid complex or hypothalamus activation are abolished after electrolytic lesions in the PAG (7).

The PAG is located in the mesencephalic region surrounding the aquaeductus Sylvii, and it is connected to multiple forebrain targets involved in the control of defensive behavior (8-10). Indeed, numerous studies confirm a critical role played by the PAG on autonomic responses (11), nociception (1,12,13), and behaviors such as flight, vocalization, and tonic immobility (1,14,15).

Functionally, the PAG is a heterogeneous structure that can be subdivided into four longitudinal columns oriented in the rostrocaudal axis: dorsomedial, dorsolateral, lateral, and ventrolateral columns (16). Previous studies have shown that different forms of emotional reactions are elaborated by distinct regions of the PAG (17,18). In this way, evidence suggests that active circa-strike behavior, such as flight and fight, are controlled by the lateral PAG, and freezing and immobility are controlled by the ventrolateral PAG (16,19).

In the laboratory, the brain circuit underlying defensive behaviors is generally studied by various models of aversive stimuli exposure, anxiogenic drugs, and electric and optogenetic stimulation (9,20-22). However, reports based on aversive stimuli that are ethologically relevant to induce innate fear- or panic attack-like responses are lacking. In this study, we hypothesized that there is a specific recruitment of PAG neurons from different columns under different conditions where either an unconditioned or a conditioned stimulus activates this structure. We tested this hypothesis by measuring the immunolocalization of Fos protein in different PAG columns of guinea pigs exposed to the prey-*vs*-snake paradigm and aversive experimental context.

## Material and Methods

### Animals

Guinea pigs (*Cavia porcellus*; Rodentia, Caviidae), n=14, weighing 450-500 g, were obtained from the animal facility of the University of São Paulo at Ribeirão Preto campus. The animals were housed in a room at 24±1°C, with a 12-h light/dark cycle (lights on at 6 am), five animals per polypropylene box (56×17×39 cm) lined with shavings, with free access to water and food throughout the experiment. The maintenance of the animals and all the procedures followed the international ethical guidelines that regulate the use of animals in the laboratory recommended by the Conselho Nacional de Controle de Experimentação Animal, Ministério da Ciência e Tecnologia (Brazil), and the study had the approval of the Committee for Animal Care and Use of the University of São Paulo (CEUA 05.1.84.53.7), campus of Ribeirão Preto. The snakes used in the experiments were wild *Boa constrictor constrictor* (Reptilia, Boidae) snakes weighing 10-12.365 kg (n=3), from the Amazon equatorial rainforest. Before experiments started, the snakes were maintained in a walled sun-lit field with appropriate shelter, grass, and water sources in the Laboratory of Neuroanatomy and Neuropsychobiology of the Ribeirão Preto Medical School of the University of São Paulo (LNN-FMRP-USP)/Behavioral Neurosciences Institute (INeC) ophidiarium, licensed by the Brazilian government (IBAMA processes 3543.6986/2012-SP and 3543.6984/2012-SP) and by the São Paulo State government (Secretaria do Meio Ambiente do Estado de São Paulo (SMA)/Departamento de Fauna (DeFau) process 15.335/2012; Mechanisms of Defensive Behaviour and Unconditioned Fear-induced Antinociception in Snake-threatened Animals (MEDUSA) Project, Sistema de Autorização e Informação em Biodiversidade (SISBIO) authorization for activities with scientific purposes process 41435-1; Sistema Integrado de Gestão Ambiental (SIGAM) authorization of installation process 39.043/2017; and SIGAM authorization for use and handling of wild snakes (process 39.044/2017). The enclosure was kept on a 12-h light/dark cycle (lights off 7:00 am) at a constant room temperature (27±1°C, 60-70% humidity). The snakes were fed with the species under study 24 h before the experiments in the same apparatus in which the experiments were performed. After the experiments, the snakes were fed, submitted to quarantine, and kept in the LNN-FMRP-USP/INeC Ophidiarium.

### Behavioral procedure

**Figure 1:**
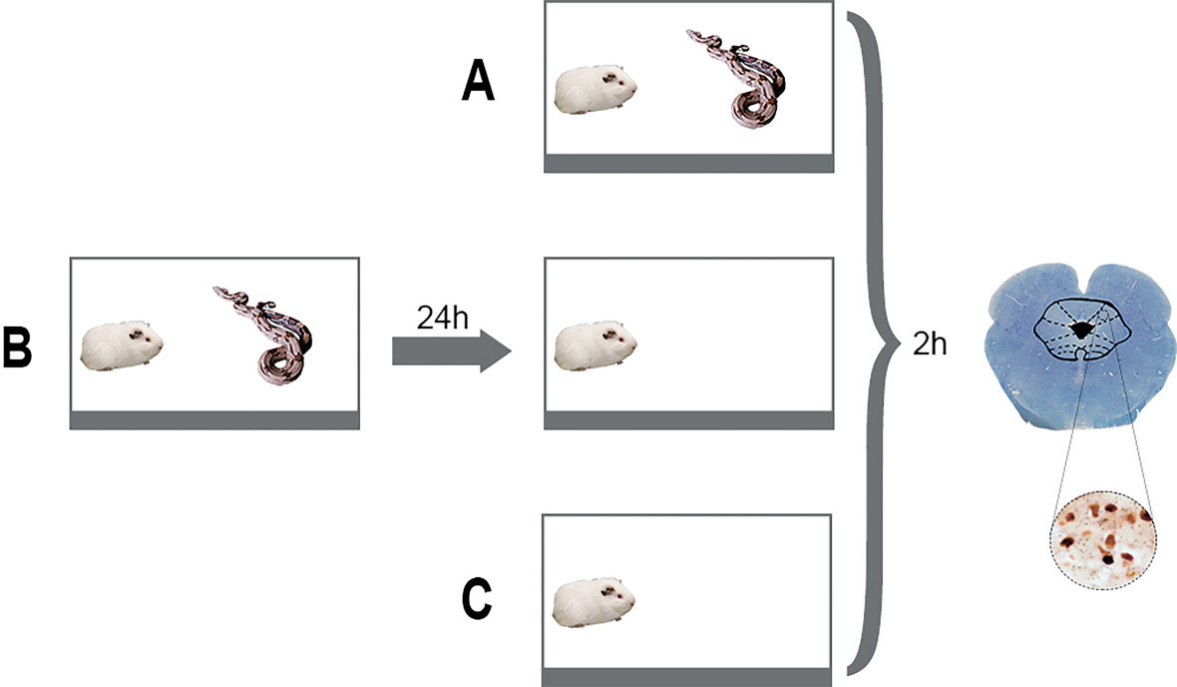
Experimental protocol. Confrontation between a guinea pig (*Cavia porcellus*) and a South American Boidae snake (*Boa constrictor constrictor)* for 15 min in the polygonal arena for snake panic test. **A**, Unconditioned fear: exposure of the guinea pig to the snake; (**B**) Conditioned fear: exposure of the guinea pig to the experimental context of confrontation 24 h after snake exposure; (**C**) Control: exposure of guinea pig to the polygonal arena.

A semi-transparent acrylic enclosure consisting of a polygonal arena (154×72×64 cm) with inner walls that were covered with a reflective film, which provided 80% light reflection to minimize visual contact between the predator and the surrounding experimental area and forced the animal to focus its attention on its prey, was used for the prey-predator confrontations (23). A red fluorescent line (4.2-mm width; Pritt Mark-It, Germany) was used to divide the arena into 20 equal rectangles. The acrylic base of the arena was placed over a rectangular stainless steel platform, and the entire apparatus was elevated on a granite rock surface (2×85×170 cm) positioned 83 cm above the laboratory floor to minimize vibratory stimuli. The polygonal arena was located in a room with controlled temperature and without sound, illuminated by three fluorescent lamps of 40 W placed on the apparatus. The experiment was performed at night. The confrontation was performed inside the polygonal arena, and the guinea pigs were placed at a distance of approximately 95 cm from the snake for 15 min (Figure 1A and B). We divided the prey animals into three groups: exposure to the predator (unconditioned fear, n=6), exposure to the context of confrontation, in which the guinea pig was exposed to the snake the day before (conditioned fear, n=4), and control group (n=4), in which the guinea pigs were maintained for 15 min in the polygonal arena under the same environmental conditions. A time-line of the experiment is shown in Figure 1.

### Immunochemistry

Two hours after the start of the experimental behavioral procedure, the animals were anesthetized with 10% ketamine (225 mg/kg; *im*) associated with 2% xylazine (30 mg/kg) and then perfused transcardially with 0.05 M phosphate-buffered saline (PBS), followed by 0.1 M sodium phosphate buffer (PB) containing 4% paraformaldehyde. The brain of each *Cavia porcellus* was removed, post-fixed for 3 h, frozen in cold isopentane (-40°C) of carbonic ice, and cut in serial sections at a thickness of 40 μm in a cryostat (CM 1950 Leica, Germany). Tissues were successively washed and incubated overnight with rabbit anti-Fos protein polyclonal antibody (SC 52, Santa Cruz Biotechnology, USA) at a concentration of 1:2000 in PBS + (0.1M PBS with 0.2% Triton-X and 0.1% bovine serum albumin, BSA). Then, histological sections were processed using the avidin-biotin-immunoperoxidase method (Vectastain ABC Kit, Vector Laboratories, USA), and Fos protein immunoreactivity was revealed by the addition of chromogen 3,3"-diaminobenzidine (DAB: 0.02%, Sigma-Aldrich, USA) at 1% hydrogen peroxide. The polyclonal anti-Fos protein antibody was omitted from negative controls. Sections were washed in PBS, mounted on gelatin slides, dehydrated through a series of ethanol and xylol solutions, glued to the coverslips, and analyzed under a light motorized photomicroscope (AxioImager Z1, Zeiss, Germany).

### Cell counts

For quantification of Fos protein immunoreactivity (Fos-IR), the anatomical localization was performed by comparing representative sections stained with cresyl violet to a stereotaxic atlas for guinea pigs (24). To describe the distribution pattern of Fos-labeled cells in the PAG, we employed the designations proposed in the Paxinos and Watson Atlas (25) from the rostral (bregma -6.6 to -7.92 mm) and the caudal levels (bregma -8.04 to -8.40 mm), which are the dorsomedial (dm), dorsolateral (dl), lateral (l), and ventrolateral (vl) columns. We considered that the nucleus of the neuron had to have an appropriate size (neuron diameter approximately 8-15 µm) and shape (oval or round). Three consecutive sections of each PAG level (rostral and caudal) were quantified for each guinea pig. In each section, the number of Fos protein-labeled neurons was unilaterally counted in the area by an experimenter blind to the treatment. A light microscope with a 10× objective was used. Fos protein-labeled neurons were counted using an image analysis system (ImageJ, NIH, USA). The number was standardized for a tissue area of 0.2 mm^2^, and the mean was calculated for each guinea pig.

### Statistical analysis

The results in each PAG column are reported as means±SE and were analyzed by two-way ANOVA, with PAG columns and groups (unconditioned fear, conditioned fear, and control) as factors for each distinct rostrocaudal level of the PAG. Additionally, a two-way ANOVA was applied to each PAG column with groups (unconditioned fear, conditioned fear, and control) and rostrocaudal levels (rostral and caudal) of the PAG as factors. Newman-Keuls' and Bonferroni's *post hoc* tests were used to determine differences between area and treatment with the level of significance set at P<0.05.

## Results

**Figure 2:**
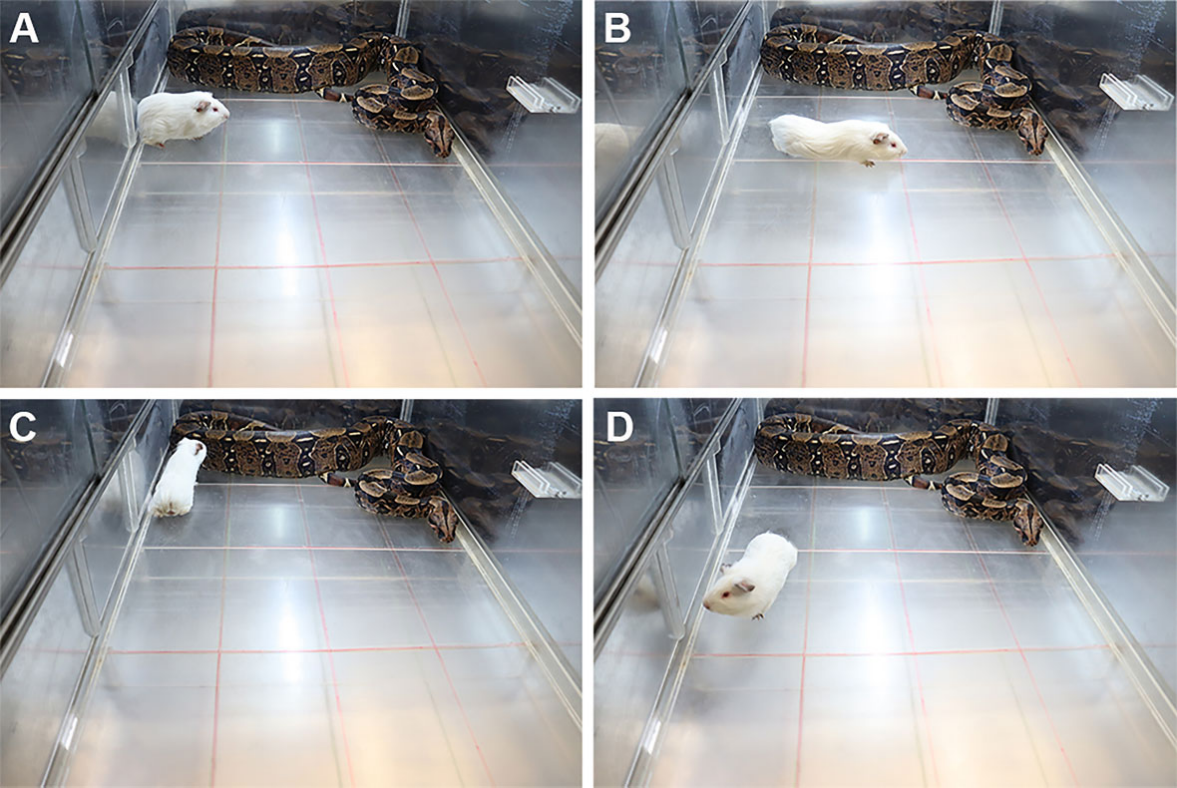
Antipredatory defensive behaviors displayed by *Cavia porcellus* threatened by non-venomous constrictor snakes (*Boa constrictor constrictor*) in the polygonal arena for the snake panic test. **A**, Robust and long-lasting defensive immobility (freezing); **B**, some additional short incidence of flat back approach; **C**, interaction with the predator; and **D**, escape.

The exposure of *Cavia porcellus* to a snake (Figure 1A, and Figure 2) elicited an antipredatory defensive behavior in the prey including defensive immobility (freezing), the most robust and long-lasting defensive behavior elicited (Figure 2A), the risk assessment response of flat back approach (Figure 2B), interaction with predator (Figure 2C), and short episodes of escape behavior (Figure 2D), followed by post-escape long-lasting freezing behavior. The re-exposure of prey to the experimental context of confrontation, without the predator, 24 h after exposure to the snake (Figure 1B), elicited a freezing response throughout the duration of the experiment (15 min). Guinea pigs exposed only to the polygonal arena (Figure 1C), without prior exposure to the predator or the aversive context, did not show freezing behavior or other defensive responses. These behavioral findings were previously reported by Leite-Panissi et al. (26). The same reactions have been displayed by rats, Syrian hamsters, and mice exposed to either venomous (5,22,23,27) or constrictor snakes (23,28) in polygonal arenas. These snakes have been ethologically validated as experimental models of either panic attack (5,23) or post-traumatic stress disorder (29).

**Figure 3:**
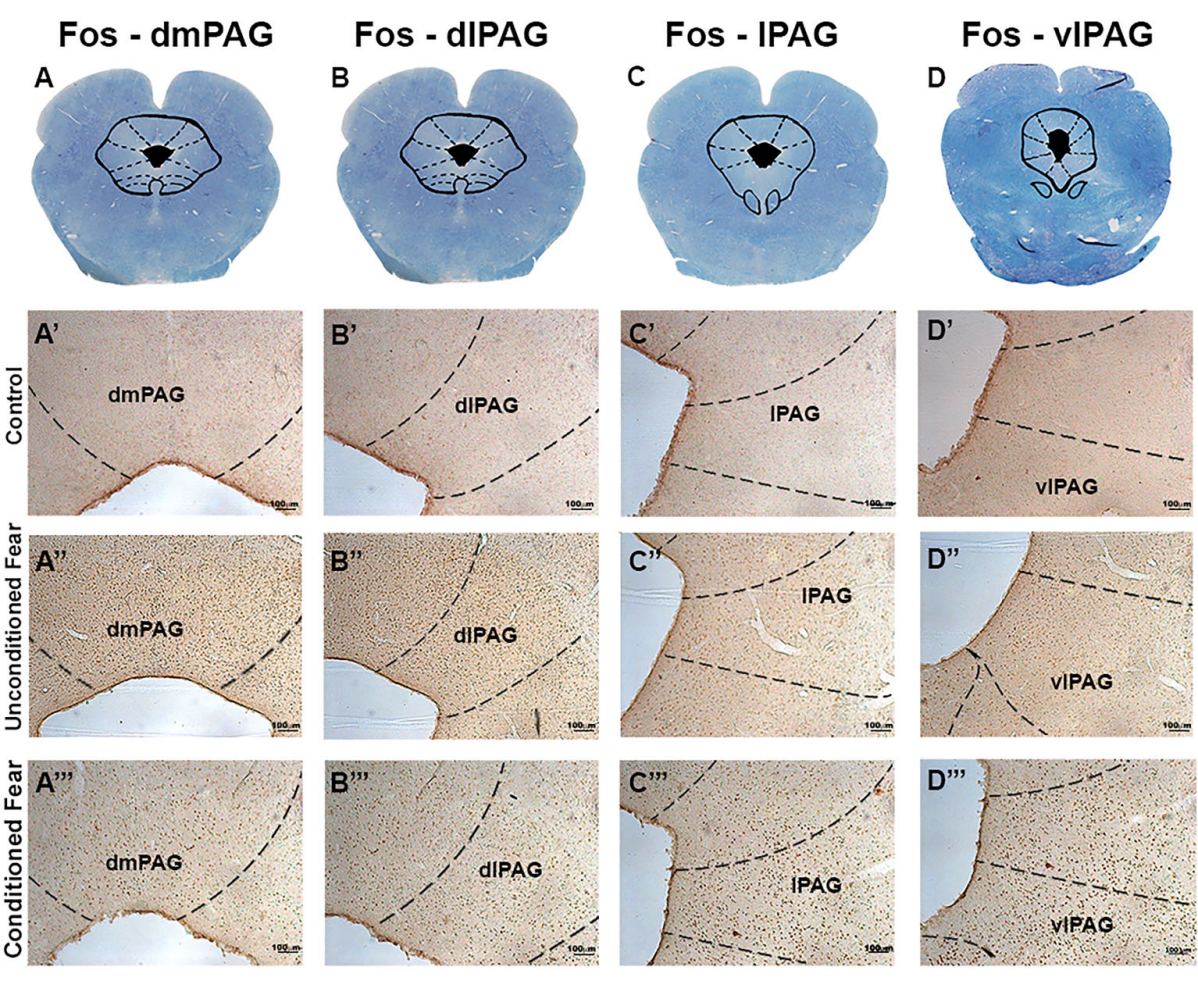
Columns of periaqueductal gray matter (PAG) were analyzed for neuronal activation during unconditioned fear (exposure to predator), conditioned fear (exposure to the experimental context 24 h after exposure to predator), and control procedure (exposure to the polygonal arena). **A**-**D**, Transverse sections of the *Cavia porcellus* midbrain at different levels of the rostro-caudal axis (Klüver-Barrera staining method). Photomicrographs of transverse sections of *C. porcellus* PAG at dorsomedial (dm) (**A'**-**A'''**), dorsolateral (dl) (**B'**-**B'''**), lateral (l) (**C'**-**C'''**), and ventrolateral (vl) (**D'**-**D'''**) columns of a representative guinea pig from control, unconditioned fear, and conditioned fear groups, showing Fos protein-labeled neuronal nuclei (brown puncta). Scale bars: 100 µm.

**Figure 4:**
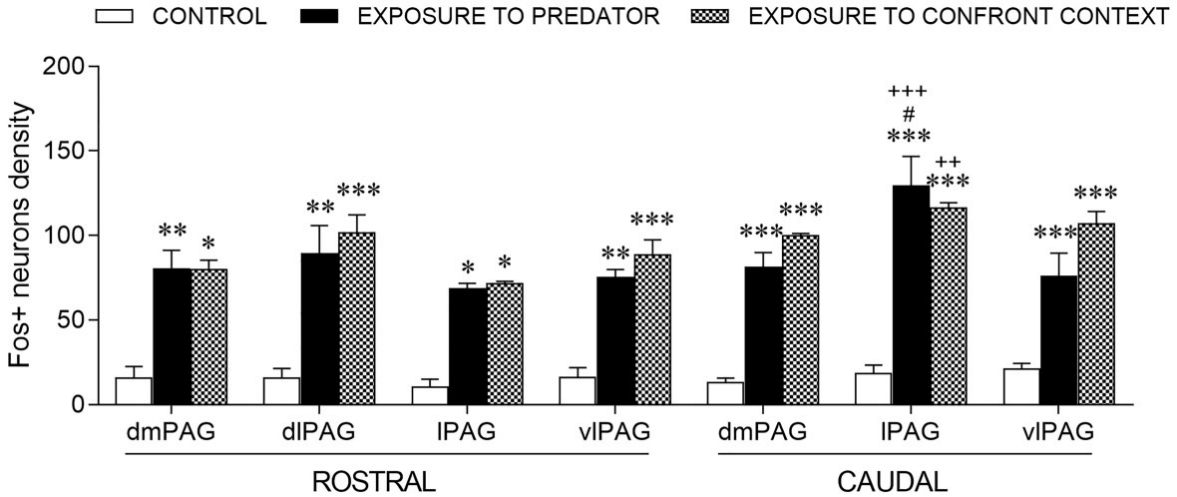
Different activation of the periaqueductal gray matter (PAG) in unconditioned and conditioned fear. Graph shows Fos protein-immunoreactivity density (Fos^+^ neurons/0.2 mm^2^) in: dorsomedial (dmPAG), dorsolateral (dlPAG), lateral PAG (lPAG), and ventrolateral columns (vlPAG) at rostral and caudal levels of the midbrain of guinea pigs eliciting unconditioned fear (exposure to the constrictor snake), conditioned fear (exposure to the experimental context), or control group (exposure to the polygonal arena). Data are reported as means±SE. *P<0.05, **P<0.01, ***P<0.001, compared to the control group (two-way ANOVA followed by Newman-Keuls' *post hoc* test). ^#^P<0.05 compared to vlPAG and dmPAG after exposure to predator. ^++^P<0.01, ^+++^P<0.0001 compared to rostral lPAG after the confrontation context or exposure to predator, respectively (two-way ANOVA followed by Bonferroni's *post hoc* test).

Analysis of Fos-IR data revealed that both conditioned and unconditioned fear paradigms promoted a robust activation of the PAG when compared to the control group (Figure 3). There was a significant effect (at the rostral midbrain level, Figure 4) of treatment (F_2,37_=55.32; P<0.0001), but not of PAG column (F_3,37_=1.40; P>0.05), nor PAG column × treatment interaction (F_6,37_=0.283, P=0.941). Exposure to the predator or to aversive experimental context increased Fos-IR expression in all columns of the PAG compared to the control group (P<0.05), but there were no significant differences among PAG columns (P>0.05).

At caudal level (Figure 4), there was a significant effect of the PAG column (F_2,20_=5.767; P=0.01), treatment (F_2,20_=104.9; P<0.0001), and the PAG column × treatment interaction (F_4,20_=3.298; P=0.03). Exposure either to the predator or to the aversive experimental context increased Fos-IR in all PAG columns compared to the control group (P<0.05). However, in contrast to the rostral level, exposure to the constrictor snake resulted in a higher Fos-IR in the lPAG than in the vlPAG and dmPAG (P<0.05). Moreover, no difference was observed in Fos-IR expression in PAG columns of the group exposed to the aversive experimental context (Figure 4).

No significant effect was found among the PAG columns when comparing unconditioned and conditioned fear paradigms in both rostral and caudal levels. Moreover, the two-way ANOVA applied to each PAG column resulted in a significant effect of treatment (F2,16=91.37; P<0.0001), PAG level (F1,16=36.84; P<0.0001), and treatment × PAG level interaction (F2,16=7.19; P=0.0059). Bonferroni's *post hoc* test revealed that the lPAG at the caudal level had a higher Fos-IR than at the rostral level in the groups exposed to the predator and to the aversive context (Figure 4).

## Discussion

Prey exposure to snake-related aversive stimuli activates a defensive-related neural network that includes the PAG in both *Mesocricetus auratus* and *Rattus norvegicus* (5,6). Here, exposure of *Cavia porcellus* to a Boidae snake and to the aversive experimental context activated all PAG columns currently studied. Moreover, at the caudal level, we found that exposure of *Cavia porcellus* to the predator caused a higher increase in Fos-IR in the lPAG compared to vlPAG and dmPAG. The PAG consists of a structure closely related to integrating somatic and autonomic responses typical of defensive behaviors (14,30,31). Our results are consistent with previous studies showing the participation of PAG in triggering defensive responses during exposure to predators as well as the aversive context of exposure (32-34).

The exposure of rodents to their natural predators has been suggested to elicit defensive behaviors related to fear and/or anxiety due to the recruitment of structures involved in limbic neural circuits (5,6). Our findings suggested a different activation pattern during unconditioned and conditioned fear. Indeed, previous studies suggest that the lPAG is associated with the modulation of fear responses to proximal danger situations, such as flight and fight behaviors (35), whereas the vlPAG is associated with inhibitory behavior (36). Moreover, the recruitment of the PAG columns seems to alternate between the rostrocaudal levels of that midbrain structure. For example, Canteras et al. (33) showed greater activation of the dPAG and lPAG columns in the rostral than in the caudal division in rats exposed to a cat, whereas Carrive et al. (37) found a more robust activation in the vlPAG after re-exposure to the aversive context. However, in the present study, a significant difference was not found in the PAG columns activation when comparing prey exposed to the predator and prey exposed to the aversive experimental context.

Although there is a large number of studies showing the triggering of different neural circuits in the PAG during both predator confrontation and exposure to predator-related stimuli (odor or exposure to the predator-related aversive environment), some results regarding the PAG columns recruited in each situation are controversial. In this perspective, Paschoalin-Maurin et al. (5) demonstrated that exposure of Syrian hamsters to the South American venomous coral snakes caused higher Fos protein expression in the dlPAG and dmPAG at the rostral and caudal levels. However, in the study by Comoli et al. (34), the exposure of rats to a natural predator caused intense activation of the dlPAG and dmPAG at the rostral level and higher activation of the lPAG and vlPAG at the caudal level. In addition, studies by Vieira et al. (38) demonstrated that guinea pigs when subjected to tonic immobility, an innate fear-related behavioral response displayed by prey in critical prey-predator situations, show higher Fos-IR in the ventrolateral column of the PAG at both rostral and caudal levels. Functional anatomical analysis of brain regions responsible for elaborating defensive behaviors has shown that the same limbic structure can have different activation patterns (8). In this context, Leite-Panissi et al. (26) demonstrated that the amygdaloid complex has different Fos-IR activation patterns in guinea pigs after unconditioned or conditioned stimuli. Specifically, the medial nucleus of the amygdala had the highest density of Fos protein-labeled neurons compared to the other amygdala nuclei after the exposure to a predator and re-exposure to the aversive context (26).

Since PAG columns are involved in distinct behavioral and physiological responses, activation of these columns might be associated with changes in the activation patterns of other brain regions involved in triggering various defensive behavioral responses. Further, it has been shown that the PAG can either stimulate or inhibit the pre-respiratory (pre-I) neurons of the pre-Bötzinger complex in a phasic and tonic manner, promoting a respiratory rhythm (39). For example, while the stimulation of dPAG and lPAG increases the firing of pre-I neurons resulting in tachypnea and inspiratory apneusis, respectively, the vlPAG stimulation promotes inhibition of pre-I neurons and diaphragm leading to apnea. These breathing patterns are consistent with the defensive responses emitted during the stimulation of different PAG columns, as dlPAG and lPAG stimulation triggers vigorous defensive responses such as flying and jumping, which requires a large amount of energy. On the other hand, vlPAG stimulation produces passive responses such as freezing and tonic immobility, and apnea allows that the prey makes as little movement as possible (13,16,39). Reinforcing that information, both chemical stimulation of N-methyl-d-aspartate (NMDA) receptors (21) and GABA_A_ receptor blockade in the dlPAG (40) elicit panic attack-like defensive responses such as freezing, running, and jumping. In addition, irreversible neurochemical lesion of the vlPAG, rather than dPAG, reduces the duration of tonic immobility, another innate fear-related defensive response (13).

During imminent risk of death or exposure to a dangerous environment, encephalic defense circuits are activated. However, the triggering of certain defensive behaviors involves the predominant activation of a specific neural system responsible for detecting and evaluating the nature of the threatening stimuli and establishing decision-making behavior to choose the most strategic reaction. The present results suggest that the activation patterns of the PAG columns differed in intensity during unconditioned and conditioned fear in guinea pigs. It is possible to hypothesize that the specific recruitment of each PAG column depended on the nature of the threatening stimulus.
